# Impacts of Industrial Restructuring and Technological Progress on PM_2.5_ Pollution: Evidence from Prefecture-Level Cities in China

**DOI:** 10.3390/ijerph18105283

**Published:** 2021-05-16

**Authors:** Ning Xu, Fan Zhang, Xin Xuan

**Affiliations:** 1 Key Laboratory of Land Surface Pattern and Simulation, Institute of Geographic Sciences and Natural Resources Research, Chinese Academy of Sciences, Beijing 100101, China; xuning_sdnu@163.com; 2 College of Geodesy and Geomatics, Shandong University of Science and Technology, Qingdao 266590, China; xuanx@sdust.edu.cn

**Keywords:** PM_2.5_ pollution, industrial restructuring, technological progress, spatial dynamic panel model, prefecture-level cities

## Abstract

PM_2.5_ pollution has produced adverse effects all over the world, especially in fast-developing China. PM_2.5_ pollution in China is widespread and serious, which has aroused widespread concern of the government, the public and scholars. This paper evaluates the evolution trend and spatial pattern of PM_2.5_ pollution in China based on the data of 281 prefecture-level cities in China from 2007 to 2017, and reveals the pollution situation of PM_2.5_ and its relationship with industrial restructuring and technological progress by using spatial dynamic panel model. The results show that China’s PM_2.5_ pollution has significant path dependence and spatial correlation, and the industrial restructuring and technological progress have significant positive effects on alleviating PM_2.5_ pollution. As a decomposition item of technological progress, technical change effectively alleviates PM_2.5_ pollution. Another important discovery is that the interaction between industrial restructuring and technological progress will aggravate PM_2.5_ pollution. Finally, in order to effectively improve China’s air quality, while advocating the Chinese government to pursue high-quality development, this paper puts forward a regional joint prevention mechanism.

## 1. Introduction

In recent years, the development model at the expense of the environment has weakened the sustainability of China’s economy. The emission of particulate pollutants in China has increased rapidly [1]. Among them, PM_2.5_ can significantly reduce the visibility of the atmosphere and is one of the main pollutants that lead to the decline of air quality.

PM_2.5_ refers to particles with an aerodynamic diameter of 2.5 μm or less, of which 1 μm is equal to 1 × 10^−6^ m [2]. In 2013, nearly 80 million people in China deviated from the normal life and production track due to PM_2.5_ pollution, and the social order was impacted [3]. Some studies have confirmed that PM_2.5_ can directly reach human lungs, thus causing great harm to human health [4]. Alleviating PM_2.5_ pollution and reducing the health hazard of PM_2.5_ on public health have become primary tasks for the Chinese government. 

Out of the responsibility to the public and the need of scientific research, scholars carried out a series of related studies. Since the 21st century, with the rapid deterioration of global air quality, a large number of related studies on PM_2.5_ pollution have emerged. Scholars have studied the diffusion and influence of PM_2.5_ from the meteorological perspective in the past time [5,6,7]. In recent years, more and more attention has been paid to the expected role of socioeconomic factors in PM_2.5_ pollution. Ji et al. (2018) conducted a study of 79 developing countries. The results based on the data from 2001 to 2010 show that the increase in income level has a positive impact on PM_2.5_ pollution, and this impact is different in stages under the influence of the level of urbanization [8]. Wang et al. (2018) explored the driving factors of PM_2.5_ pollution in G20 countries. The empirical results show that political globalization has a significant impact on high-and low-polluting countries, while the existence of democracy has a weak impact on low-polluting countries. Furthermore, the results based on the mediating effect test show that the per capita GDP is the bridge between the above two indicators on PM_2.5_ pollution [9]. Mania et al. (2020) used an air sampler to quantitatively monitor the concentration of PM_2.5_ in Pacific island countries. At the same time, the results of factor analysis show that the increase in the number of second-hand cars and lax emission restrictions are the main factors contributing to the aggravation of PM_2.5_ pollution in Pacific island countries [10]. Aiming at the serious fine particulate pollution in North China, Zhang et al. (2020) explored the impact of a series of socioeconomic indicators on PM_2.5_ pollution. The results show that the social transformation characterized by urbanization and intensive traffic network significantly aggravates the development of PM_2.5_ pollution, and the development of service industry can effectively alleviate PM_2.5_ pollution [11]. The situation of PM_2.5_ pollution in provincial and municipal areas has also attracted more and more attention. For example, in a study on Beijing, Xu et al. (2020) captured the positive impact of government regulation on improving PM_2.5_ pollution, providing a basis for the rationality and effectiveness of government control of air pollution [12]. Lin et al. (2020) took 255 cities in China as the study samples and collected data from 2000 to 2015, exploring the relationship between forest and PM_2.5_ pollution. The results show that increasing forest area can alleviate PM_2.5_ pollution, specifically, 1% increase in forest area can reduce PM_2.5_ concentration by 2.53% [13].

On the other hand, the methodology of PM_2.5_ pollution research is also worthy of attention. The existing quantitative research paradigm can be roughly divided into two categories. One is to judge the influencing factors of PM_2.5_ pollution based on the correlation test. For example, Zhang et al. (2015) used multifractal asymmetric detrended cross-correlation analysis to measure the correlation between various meteorological factors and PM_2.5_ pollution. The results show that the increase of temperature, air pressure, relative humidity and wind speed can significantly reduce the local PM_2.5_ concentration [14]. Based on annual cross-sectional data and correlation analysis, Huang et al. (2019) judged that the increase of household income and education level can effectively alleviate PM_2.5_ pollution [15]. The second kind of analysis path is quantitative analysis based on the parameterized econometric regression model. For example, Zhao et al. (2019) used the ordinary least squares (OLS) method to explore the impact of various socioeconomic indicators on PM_2.5_ pollution [16]. In addition, panel quantile regression model [9], multi-objective ensemble model [17] and STIRPAT model [18,19] are widely used. By using the above research methods, the influence of a certain index on PM_2.5_ pollution can be obtained from different angles, and the degree of fitting of fit is high. However, this is not enough to explain the complex mechanism of the development of PM_2.5_ pollution. According to the first law of geography, everything is related to other things, and neighboring things are more closely related [20]. PM_2.5_ is easy to diffuse [21], and PM_2.5_ pollution in neighboring areas may have spatial correlation. If the spatial relationship of data is not extracted and processed, it will lead to the potential spatial autocorrelation problem, which will eventually lead to the distortion of the model results [22]. In other words, the macro impact analysis of PM_2.5_ needs to be improved by spatial methods. In reality, due to the slow adjustment of emission reduction technology, environmental policy and inertia of other economic factors, the adjustment of pollution emission is often a continuous and slow dynamic process. The static model will lead to estimation deviation due to ignoring the dynamics of the target variable, which will eventually affect the authenticity of the economic explanation of the estimation coefficient of the explanatory variable [23]. Considering that PM_2.5_ pollution may have the above two properties, a method combined with time and space dynamics is needed to better simulate the actual situation of PM_2.5_ pollution.

At the present stage, for China, industrial structure adjustment and technological progress are the fundamental driving force to alleviate PM_2.5_ pollution. The Chinese government is committed to adjusting the industrial structure at the present stage, and these reform measures have been proved to affect the air quality [13,20]. At present, most scholars focus on the relationship between industrial restructuring and PM_2.5_ pollution in China. The general view of existing research is the upgrading the industrial structure from the secondary industry to the tertiary industry is a valid path to solve the problem of PM_2.5_ pollution [11,24,25]. During the 12th five-year Plan and 13th five-year Plan period, Chinese governments at all levels actively advocated strengthening technological transformation and upgrading in the fields of production and consumption, developing new technologies, processes and equipment for energy conservation and environmental protection, and giving full play to the leading role of scientific and technological innovation in green development [26,27]. Up to now, the research on the impact of technological progress on atmospheric environment has focused on energy consumption and greenhouse gas emissions [28,29]. In recent years, the diffusion intensity of PM_2.5_ pollution in Chinese mainland region has been restrained to a certain extent, which is intrinsically related to the changes in technology and industry. However, from the perspective of quantitative analysis, the specific effects of technical factor and industrial factor on alleviating PM_2.5_ pollution have not been deeply explored.

Do technological progress and industrial structure adjustment restrain PM_2.5_ pollution? Considering the relevance and compatibility of production and technology, what is the combined effect of the two on PM_2.5_ pollution? Meanwhile, prefecture-level cities are administrative divisions in China, which rely on huge energy consumption to maintain their development and support a large number of people. At the same time, energy consumption has been proved to be the main driver of deteriorating PM_2.5_ pollution [30]. Therefore, cities are both producers and main victims of PM_2.5_ pollution [31]. Therefore, in order to answer the above two questions, this paper selects the data of 281 prefecture-level cities, explores the dynamic evolution characteristics and geographical distribution pattern of China’s PM_2.5_ pollution, and focuses on the effect of industrial structure adjustment and technological progress on PM_2.5_ pollution. The marginal contributions that may be made in this paper are as follows: (1) From the research perspective, this paper takes both technological factor and industrial factor as the core part of the PM_2.5_ pollution research framework; (2) In terms of research methods, taking the dynamic characteristics and spatial correlation characteristics of PM_2.5_ pollution diffusion as the entry point, this paper establishes the spatial dynamic panel model to explore how technological progress and industrial structure adjustment affect PM_2.5_ pollution.

The rest of this paper is arranged as follows: Section 2 introduces the methodology and data sources of this paper. Section 3 contains the development of PM_2.5_ pollution in China. Section 4 is the results of quantitative analysis based on the model. Section 5 contains the main conclusion and related discussion.

## 2. Methodology and Materials

### 2.1. Model Derivation

Grossman and Krueger put forward an analytical framework for the environmental effects of trade, and decomposed the environmental effects of international trade into three aspects: structural effects, technological effects and scale effects [32]. This paper improves the above framework to identify the influencing factors of PM_2.5_ pollution. The improved benchmark framework is as follows:

(1) $${PM}_{it}=f\left({ISU}_{it}{,\;ISR}_{it}{,\;TP}_{it}{,\;Z}_{it}\right)$$

In this framework, *i* stands for prefecture-level city, t stands for year, and the interpreted variable *PM* stands for PM_2.5_ concentration, which is used to measure the degree of PM_2.5_ pollution. *ISU* stands for upgrading of industrial structure, *ISR* stands for rationalization of industrial structure, *TP* stands for technological progress, and *Z* is the set of control variables. Considering that PM_2.5_ pollution in prefecture-level cities may have temporal dynamics, this paper establishes the following regression model:

(2) $${PM}_{it}{=A+\tau PM}_{i(t-1)}{+\beta }_{1}{ISU}_{it}{+\beta }_{2}{ISR}_{it}{+\beta }_{3}{TP}_{it}{+\alpha Z}_{it}{+\varepsilon }_{it}$$

Equation (2) is a general dynamic panel model, *A* is a constant term, *β*_1_-*β*_3_ represent the estimated coefficients of the core explanatory variables, and α includes the estimated coefficients of the control variables, and *ε* is a random interference term. *τ* is the first-order lag estimation coefficient of the explained variable, which can reflect the influence of previous PM_2.5_ pollution on current PM_2.5_ pollution. It is worth noting that the research unit of this paper is the city, which forms a spatial relationship that cannot be ignored with the flow of elements as the link. Focus on the spatial correlation of PM_2.5_ pollution, this paper establishes the following regression model:

(3) $$\begin{array}{l} {PM}_{it}{=A+\beta }_{1}{ISU}_{it}{+\beta }_{2}{ISR}_{it}{+\beta }_{3}{TP}_{it}{+\alpha Z}_{it}+\rho \sum_{j=1}^{n}W_{ij}{PM}_{jt}{+\varepsilon }_{it}{+\nu }_{i}{+\eta }_{t}\\ {\;\varepsilon }_{it}=\lambda \sum_{j=1}^{n}W_{ij}\varepsilon_{jt}+\theta_{jt} \end{array}$$

Equation (3) is a spatial static panel model. Both *ρ* and λ are estimated coefficients of spatial correlation, which reflect the spatial lag effect and spatial error spillover effect of regional PM_2.5_ pollution. *W_ij_* is a geo-spatial inverse distance matrix based on the spatial distance relation of geographic units. We use the reciprocal of Euclidean distance between prefecture-level city *i* and prefecture-level city *j* to calculate *W_ij_*, that is, *W_ij_* = 1/*d_ij_*. *ν* stands for regional non-observed effect and *η* stands for time unobservable effect. In summary, if the dynamic effect and spatial effect of PM_2.5_ pollution are considered at the same time, the first-order lag term and spatial lag term of PM_2.5_ pollution can be included in the equation, and the following compound model can be established:

(4) $$\begin{array}{l} {PM}_{it}{=A+\tau PM}_{i(t-1)}{+\beta }_{1}{ISU}_{it}{+\beta }_{2}{ISR}_{it}{+\beta }_{3}{TP}_{it}{+\alpha Z}_{it}+\rho \sum_{j=1}^{n}W_{ij}{PM}_{jt}{+\varepsilon }_{it}{+\nu }_{i}{+\eta }_{t}\\ {\;\varepsilon }_{it}=\lambda \sum_{j=1}^{n}W_{ij}\varepsilon_{jt}+\theta_{jt} \end{array}$$

Equation (4) is a spatial dynamic panel model, which is essentially a combination of Equations (2) and (3). Seen from the model components, it can control both the dynamic characteristic and spatial characteristic of PM_2.5_ pollution.

### 2.2. Variables Description and Data

#### 2.2.1. Core Explanatory Variables

(1) Industrial structural upgrading (*ISU*): The international community usually divides various industries into three categories. This paper takes the proportion of the added value of the tertiary industry as the indicator of the upgrading of the industrial structure [33].
(2) Industrial structure rationalization (*ISR*): Theil introduced the concept of entropy in information theory into economics, and used it to measure income inequality, which better reflects the uneven distribution of income factors in regions [34]. Therefore, the Theil index can be used to evaluate the rationalization of the industrial structure. The calculation method of Theil index is as follows:

(5) $$Theil\;index=\sum_{i=1}^{n}\frac{W_{i}}{W}\ln(\frac{W_{i}}{P_{i}}/\frac{W}{P})$$

In this formula, *W* represents regional GDP, *P* represents employed population, *i* represents specific industry, and *n* represents the grouping of industrial structure. The smaller *Theil index* is, the higher the rationalization degree of industrial structure is. In order to realize the consistency between *ISR* numerical change and rationalization change direction of industrial structure, this paper carries out reciprocal transformation of *Theil index*, that is, *ISR* = 1/*Theil index*, the higher *ISR*, the higher rationalization of industrial structure.

(3) Technological progress (*TP*): This paper takes the change of total factor productivity (*TFPCH*) as the indicator of *TP*. Because there is no need to estimate parameters in advance, data envelopment analysis (DEA) can effectively reduce errors and avoid the influence of subjective factors, and is suitable for evaluating the relative effectiveness of multi-input decision-making units [35]. Therefore, DEA-Malmquist productivity index method is used to calculate the *TFPCH*. At the same time, this method can further decompose *TFPCH* into technical change (*TC*) and efficiency change (*EC*) [36]. All the calculation work is based on the index database with the time span from 2006 to 2017 and DEAP 2.1 platform. The input indicators set in this paper include capital, energy and labor. Specifically, this paper uses capital stock to represent capital investment. The former is calculated based on the price level in 2006 and the perpetual inventory method [37]; Convert all kinds of energy consumed by prefecture-level cities into standard coal and characterize energy input by this; Using the scale of employed persons to represent labor input. In this paper, the price index of 2006 is used to offset the GDP, and the processed GDP is used to characterize the output.

#### 2.2.2. Control Variables

(1) Economic development (*ED*): Most of the related researches on the correlation between economic development and PM_2.5_ pollution are based on the EKC (environmental Kuznets curve) theory [32]. This paper uses per capita GDP to measure the economic status of cities.
(2) Urbanization (*UR*): The urbanization process has changed the original natural and social conditions, and therefore, cities have become spatial containers of PM_2.5_ pollution [18]. Many studies have confirmed the positive impact of urbanization on PM_2.5_ pollution [38,39,40]. This paper uses the proportion of urban population to the total population to measure the urbanization level.
(3) Energy intensity (*EI*): The general view is that pollutants emitted by energy consumption constitute the important supplement of PM_2.5_ [41,42]. Considering the difference of city scale, energy intensity has a higher explanatory power for PM_2.5_ pollution. In this paper, all kinds of energy consumed by cities are converted into standard coal, and the energy intensity is measured by the amount of standard coal consumed per 10,000 yuan of GDP.
(4) Temperature (*TE*): Tai et al. (2010) analyzed the impact of climate change on PM_2.5_ pollution, and found that high air temperature made PM_2.5_ pollution situation worsening [43]. In this paper, the annual average temperature is used to measure the temperature of each city [9,44].
(5) Precision (*PR*): The precipitation process is an important way to remove particulate pollutants [45]. In this paper, the average value of annual precipitation recorded by urban meteorological monitoring stations is used to measure urban precipitation.

### 2.3. Data Source

The PM_2.5_ data used for analysis in this paper is the product provided by Dalhousie University Atmospheric Composition Analysis Group, and is widely used in global, national and regional multi-scale research [46,47]. Based on the ArcGIS platform, this paper analyzes the original data to obtain the PM_2.5_ concentration level of each city. Socioeconomic data comes from various issues of China City Statistical Yearbook, and statistical bulletins issued by prefecture-level cities and other public data. The energy use data of each city is based on the decomposition of provincial energy consumption data and night lighting data. Individual missing data were processed by means of imputation. The temperature and precipitation data are obtained from meteorological monitoring stations set up in various cities. In view of the completeness and continuity of the data and the adjustment of administrative divisions, this paper finally selected 281 prefecture-level cities in China as the research objects. 

**Figure 1:**
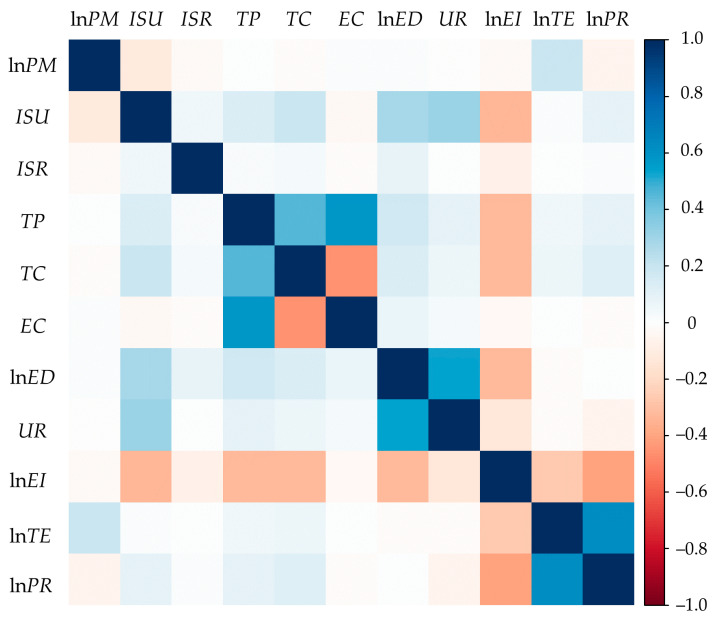
Correlation test results for the model variables.

Table 1 shows the statistical results of the variables. In the model estimation process, in addition to the variables measured in percentage units, this paper performs logarithmic transformation on all other variables to reduce the damage caused by heteroscedasticity to the accuracy of the estimation results. Multicollinearity is ubiquitous, which will increase the variance of regression coefficient and reduce the reliability of model estimation. Before the regression of the three models, the multicollinearity test was carried out in this paper. On the one hand, the correlation coefficient matrix shows that there is no highly correlated set of variables (Figure 1). On the other hand, the VIFs are all less than 5, which indicates that there is no serious multicollinearity problem. Therefore, it can be considered that the model regression carried out by large samples in this paper is in line with the standard of econometric analysis. 

## 3. Temporal-Spatial Characteristic of PM_2.5_ Pollution

### 3.1. Dynamic Evolution of PM_2.5_ Pollution 

**Figure 2:**
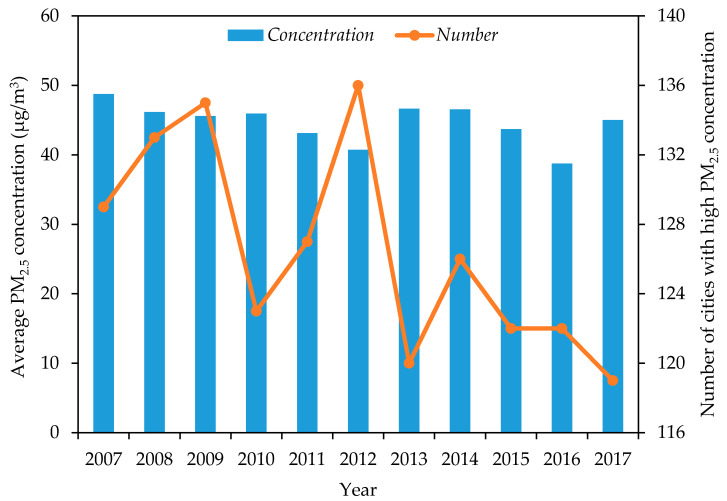
China’s PM_2.5_ pollution status from 2007 to 2017.

In order to explore the development trend of PM_2.5_ pollution, this paper calculates the annual average value of PM_2.5_ pollution and the number of cities above average in the study area from 2007 to 2017 (Figure 2). The average concentration of PM_2.5_ pollution has fluctuation and stage. From 2007 to 2012, the concentration showed a downward trend. Then, the concentration increased from 40.73 μg/m^3^ in 2012 to 46.64 μg/m^3^. Then, the concentration decreased between 2013 and 2016, however, it showed an upward trend at the end of the study period. Overall, the degree of PM_2.5_ pollution in the study area has been alleviated, although the data show that the concentration has only decreased by 7.66%. In addition, the number of above-average cities fluctuates over time, but the number is limited. During the study period, higher-than-average cities accounted for 42.35% to 48.04% of the total sample.

**Figure 3:**
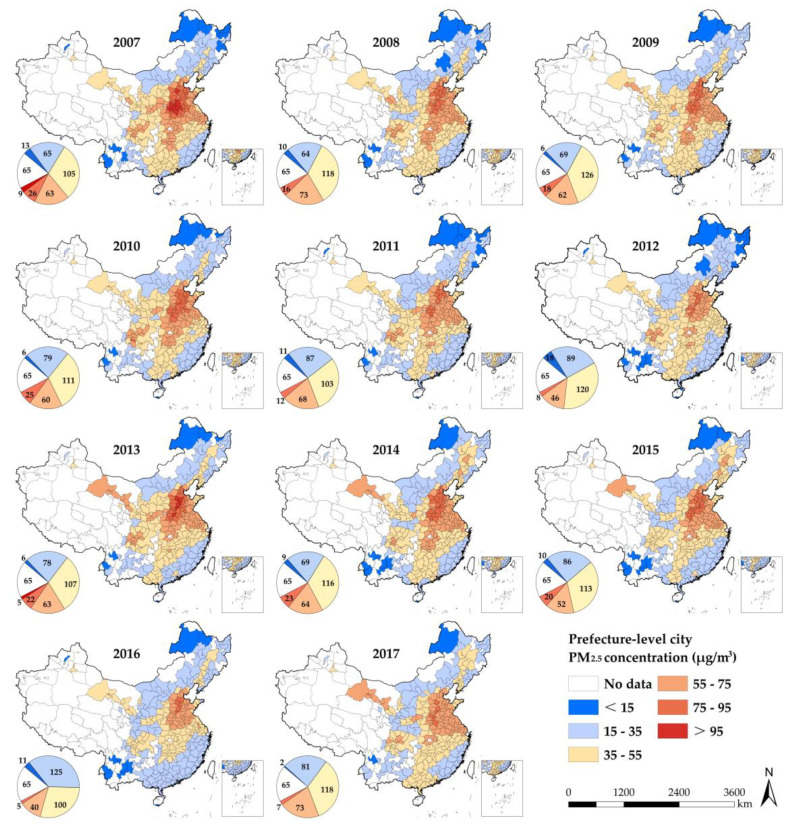
Geographical distribution of China’s PM_2.5_ pollution.

According to the “Ambient Air Quality Standard” (GB3095-2012) issued by the Chinese government, this paper divides the PM_2.5_ concentration into six intervals (Figure 3). Overall, the number of cities with PM_2.5_ concentrations lower than 15 μg/m^3^, 55~75 μg/m^3^, and 75~95 μg/m^3^ all decreased significantly, while the remaining two categories increased. China’s PM_2.5_ pollution problem has improved slightly in the past ten years. According to the distribution situation, in 2007, the high-value areas of PM_2.5_ concentration were mainly located in the border areas of North China, Central China and East China and Shandong Peninsula. Since then, the distribution of high-value regions has shown remarkable dynamic changes, and the development trend of local contraction has appeared in recent years. Low-value areas are scattered, which can be roughly divided into three zones: northeast, southwest and southeast coastal areas. An important message shown in Figure 3 is that PM_2.5_ pollution has obvious path-dependent characteristics. For a single city, this path dependence is reflected in the time series correlation of the average annual PM_2.5_ concentration.

### 3.2. Spatial Autocorrelation of PM_2.5_ Pollution 

Studies have proved that spatial autocorrelation analysis is scientific and available for atmospheric environment research [48]. Moran’s *I* is one of the spatial autocorrelation measures [49]. This paper first obtains the overall spatial distribution pattern of PM_2.5_ pollution based on the Global Moran’s *I*. The calculation method of Global Moran’s *I* is as follows:

(6) $$\;I=\frac{n\sum_{i=1}^{n}\sum_{j=1}^{n}W_{ij}{(p}_{i}-\bar{p})(p_{j}-\bar{p})}{\left(\sum_{i=1}^{n}\sum_{j=1}^{n}W_{ij}\right)\sum_{i=1}^{n}{{(p}_{i}-p)}^{2}},\;(i\neq j)$$

In the formula, *I* represent Global Moran’s *I*. pi and *p_j_* represent PM_2.5_ concentration values of cities *i* and *j*, respectively, *n* is the total number of cities. The significance test of Global Moran’s *I* can be realized by using standardized statistics *Z(I)*:

(7) $$Z(I)=\frac{I-E(I)}{\sqrt{Var(I)}}$$

In which *E(I)* and *Var(I)* are the expectation and variance of the global Moran’s *I*, respectively. Global Moran’s *I* and *Z(I)* results of PM_2.5_ pollution are shown in Table 2. Measured value is greater than 0.49 and *Z* statistic are significantly positive in each year, which indicates that PM_2.5_ pollution has significant spatial correlation and aggregation. From 2007 to 2017, Global Moran’s *I* declined, which indicated that the aggregation of PM_2.5_ pollution in China was weakened at the prefecture-level cities level.

We use Local Moran’s *I* to analyze the correlation degree between PM_2.5_ pollution in one city and PM_2.5_ pollution in neighboring cities. The method for calculating Local Moran’s *I* is as follows:

(8) $$Local\;Moran’s\;I=\frac{n\left(p_{i}-\bar{p}\right)\sum_{j=1}^{m}W_{ij}\left(p_{j}-\bar{p}\right)}{\sum_{i=1}^{n}{{(p}_{i}-\bar{p})}^{2}},\;(i\neq j)$$

**Figure 4:**
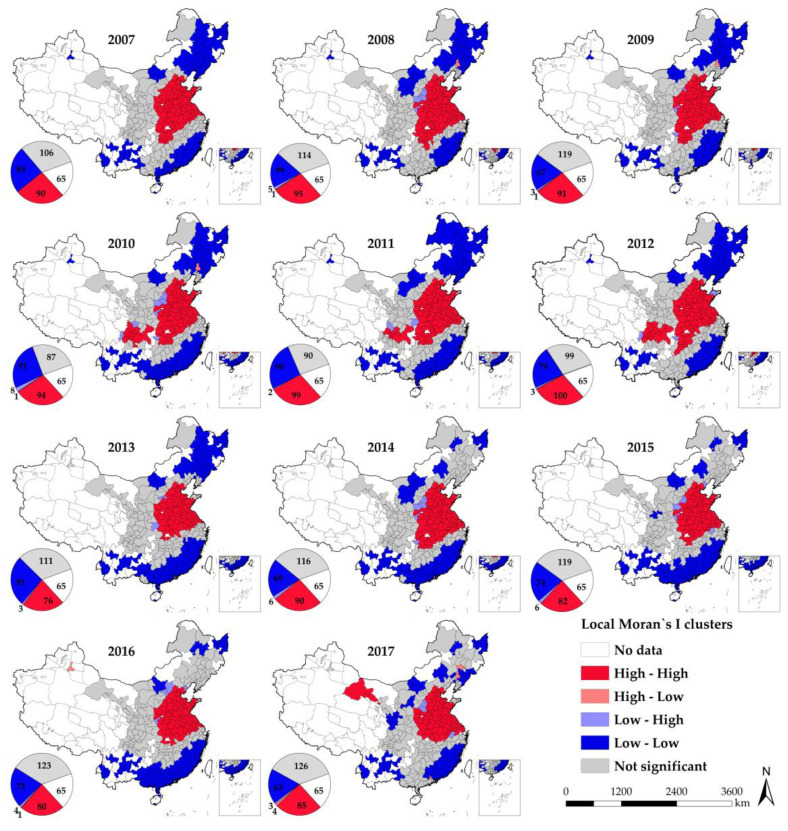
Local autocorrelation map of China’s PM_2.5_ pollution.

In which *p_i_*, *p_j_*, *n* and *W_ij_* have the same meanings as above, and m is the number of cities geographically adjacent to city *i*. Figure 4 shows the local autocorrelation results of PM_2.5_ concentration. The results of local autocorrelation are highly consistent with the geographical distribution pattern of PM_2.5_ concentration. High-High clustering is mainly located in the area of Central China and Shandong Peninsula. Northeast China, Southwest China and Southeast Coast are the concentrated areas of Low-Low clustering. There are fewer High-Low clustering and Low-High clustering. The results of local spatial autocorrelation indicate that the spatial heterogeneity of PM_2.5_ pollution distribution is small, and the regional integration of PM_2.5_ pollution is obvious.

## 4. Impacts of Influencing Factors on PM_2.5_ Pollution

### 4.1. Model Recognition

We find that PM_2.5_ pollution in China has significant temporal dynamics and spatial correlation. Therefore, compared with the two other non-general models, spatial dynamic panel model has theoretical advantages. In order to verify whether the advantages of spatial dynamic panel model can be continued in practice, this paper estimates the three models and compares the results.

It should be noted that in this study, the first-order lag term of the dependent variable is regarded as one of the explanatory variables, and the panel data has cross-sectional dependency. Therefore, using traditional estimation methods to estimate dynamic panel data will inevitably lead to the bias and inconsistency of parameter estimation, which will distort the economic meaning of inference based on parameters. In order to get consistent estimators, the widely used estimation method in related research is the difference generalized moment estimation (Difference-GMM) proposed by Arellano and Bond [50]. Unfortunately, when the steady-state level adjustment speed of the explained object is slow, the lag level tool variable used by Difference-GMM will become very weak. Blundell and Bond proposed system generalized moment estimation (System-GMM) based on Difference-GMM, which solved the above problems well [51]. In this paper, System-GMM is used to estimate the general dynamic panel data model. Kukenova and Monteiro compared many estimation methods when studying the spatial effects of dynamic panel models, and found that System-GMM has the best estimation effect among different methods. They developed the generalized moment estimation of spatial system (Spatial System-GMM) based on system-GMM [52]. Based on this, this paper uses Spatial System-GMM to estimate the spatial dynamic panel model. In addition, in order to weaken the influence of heteroscedasticity, the standard error of parameter estimation of system GMM adopts robust estimation. Finally, for the spatial static panel model, this paper uses ML method [53] to estimate. So far, this paper specifies specific estimation methods for three non-general panel models. Table 3 shows the detailed estimation results of the three models, including two different specifications of model estimation. 

Specifically, for the core explanatory variables, only their independent effects on PM_2.5_ concentration are considered in the model (*a*), model (*c*) and model (*e*). model (*b*), model (*d*) and model (*f*) consider the interaction between technological progress and industrial structure upgrading and rationalization. This can achieve intra-group comparison of the estimation results of the same model, which is a method of robustness test.

In order to judge whether the System-GMM method is specified correctly, it is necessary to carry out two sets of specification tests. First, over-recognition test should be used to judge the overall effectiveness of tool variables in GMM framework. Sargan statistics and Hansen statistics can be used for over-recognition test, but the former is not robust to heteroscedasticity [54]. Therefore, this paper only reports the Hansen statistics. The test values are all greater than 0.10, and the original hypothesis is not rejected, which indicates that there is no over-identification problem in GMM estimation of the system. Secondly, the consistency of System-GMM estimator requires that the error term of the original horizontal equation has no sequence correlation, so this paper uses AR test to test the sequence correlation of residual term. The results of AR test show that the random disturbance term has first-order sequence correlation but no second-order sequence correlation, which satisfies the requirement of moment constraint. Reasonable screening of different spatial models the key step to estimate spatial panel data. Based on the results of LM and Robust LM statistics, this paper use SAR model to estimate spatial models.

By observing Table 3 as a whole, it can be found that the estimation coefficients of models (*e*) and (*f*) are more significant than those of models (*a*)–(*d*). This is mainly because model (*e*) and model (*f*) control the spatial effect of PM_2.5_ concentration and weaken the partial deviation in the estimation process of model (*a*) and model (*b*). In the same way, model (*e*) and model (*f*) consider the dynamic effect of PM_2.5_ concentration ignored by model (*c*) and model (*d*), which makes the estimation result better. In conclusion, the spatial dynamic panel model shows its advantages in theory and practice. Therefore, this paper chooses model (*e*) and model (*f*) as the final interpretation models.

### 4.2. Analyses of Results

According to the estimation results of models (*e*) and (*f*), the estimation coefficients of the first-order lag terms are both significantly positive at 1% significance level, which indicates that the formation and adjustment of PM_2.5_ is indeed a continuous and cumulative process. Path dependence of PM_2.5_ pollution indicates that it is urgent to carry out regional PM_2.5_ pollution control work decisively. The spatial regression coefficients are greater than 0.46, and have passed the significance level of 1%, which indicates that PM_2.5_ pollution in China has spatial diffusion effect. The Chinese government should adopt a joint prevention and control strategy in the process of PM_2.5_ pollution control; otherwise, the local control work may be in vain, and the PM_2.5_ pollution crisis may come back after a period of improvement.

The estimation result of model (*e*) shows that the coefficient of industrial structure rationalization is significantly negative after controlling *ED*, *UR*, *EI*, *TE* and *PR*. This discovery highlights the importance of rationalizing industrial structure to alleviate PM_2.5_ pollution in China. The coefficient of technical change is negative at 5% significance level, while the coefficient of efficiency change is significantly positive. At the same time, the coefficient of technical change is greater than the coefficient of efficiency change. This indicates that technical change is essential for alleviating PM_2.5_ pollution. The negative effect of efficiency changes in mitigating PM_2.5_ pollution deserves Chinese government’s attention. For a long time, in order to achieve rapid economic development, China’s development model pays more attention to quantity rather than efficiency. This has led to serious problems of low production efficiency and overcapacity in China’s steel, construction and cement industries. The double effects of lack of effective administrative measures and lagging market reform make the problem of overcapacity in China’s industries always exist, and eventually lead to negative environmental effects.

The estimation results of the model (*f*) show that the estimation results become more complicated after considering the technological progress and the interactive terms of the core explanatory variables. First, the coefficient of industrial structure upgrading has changed from insignificant to significant, and the coefficient of industrial structure rationalization is more significant. From the perspective of regression parameters, for unit increase in industrial structure upgrading and industrial structure rationality, the PM_2.5_ concentration will decrease by 0.78% and 11.12%, respectively. The empirical results of Zhang et al. (2020) show that for 1% increase in the output value of the tertiary industry, the concentration of PM_2.5_ decreases by an average of 0.99% [11]. In addition, the research results of Zhang et al. (2019) show that the increase in the proportion of output value of the secondary industry will lead to the aggravation of PM_2.5_ pollution, that is, for 1% increase in proportion, the concentration of PM_2.5_ will increase by 1.03% [18]. In a word, the realistic possibility that the shift of the focus of industrial development from the secondary industry to the tertiary industry will improve air quality has been confirmed again in this study. Although the role of PM_2.5_ emission reduction of industrial structure rationalization has not been discussed in previous studies, in this study, the environmental improvement effect of industrial structure rationalization is more than ten times that of industrial structure upgrading. Therefore, full attention should be paid to the positive environmental effects contained in the process of industrial structure rationalization. Secondly, the regression coefficient of technological progress is less than 0 and has passed the significance test of 5%. This indicates that technological progress has a significant positive effect on alleviating PM_2.5_ pollution in China. If the technological progress increases by one unit, the decrease of PM_2.5_ concentration will reach 22.50%. This is similar to the empirical results of Yi et al. (2020), which affirmed the positive role played by technological progress in alleviating PM_2.5_ pollution, and pointed out that the average concentration of PM_2.5_ decreased by about 20% for every 1% increase in neutral technological progress [55]. Thirdly, the results of model (*f*) show that the interaction between technological progress and industrial restructuring promotes the development of PM_2.5_ pollution. The joint item of technological progress and industrial structure upgrading increased by 1 unit, and the average increase in PM_2.5_ concentration was 0.60%. As for the rationalization of industrial structure, it will also worsen the situation of air pollution when combined with technological progress.

In terms of control variables, economic development has a significant negative impact on PM_2.5_ pollution, which indicates that the positive externalities of economic scale have been brought into full play, and sustained economic development is conducive to improving PM_2.5_ pollution. Economic development level measured by currency can quantify the final results of urban social and economic activities, but it is difficult to show more details. In fact, there is a possibility that the upgrading of industrial structure, the improvement of energy-saving technology and the overall effect of public awareness of environmental protection offset the negative environmental effects caused by economic development. Just as EKC theory states, environmental quality will deteriorate first and then improve with economic development. The above reasons make economic development play a negative role in the development of PM_2.5_ pollution in China. According to the regression results, urbanization is beneficial to alleviate PM_2.5_ pollution, which is different from most existing studies. Urbanization plays a positive and negative role in environmental quality. The normal operation of the city needs energy maintenance. In addition, the destruction of natural environment and industrial dust caused by urban construction and expansion are also important ways for urbanization to damage environmental quality. However, what cannot be ignored is that the rapid development of large-scale production and pollution control, increasingly perfect environmental laws and regulations and high-quality citizens’ awareness of environmental protection have promoted the improvement of urban air quality. In this paper, if the inhibition effect of urbanization process on PM_2.5_ pollution deterioration exceeds the promotion effect of other factors, urbanization can show positive environmental effects. Higher energy intensity will make PM_2.5_ pollution worse. The improvement of energy intensity means the increase of power consumption. Considering the fact that most cities in China still rely on coal for power generation, the increase of power consumption indirectly leads to the generation of dust and fine particles, which makes PM_2.5_ pollution worse. The effects of natural factors on PM_2.5_ pollution are consistent with most existing research results. Under high temperature conditions, it is easier to form a stable inversion layer over cities, which leads to PM_2.5_ stably and muchly suspended in the air. Precipitation can promote the wet deposition of aerosol and realize the scouring of PM_2.5_ particles.

## 5. Discussion and Conclusions 

### 5.1. Main Conclusions

Since the reform and opening up, China has made remarkable achievements. However, China’s extensive development model has led to ecological destruction, environmental scarcity and environmental pollution. In particular, a large number of fine particles emitted from the production process have caused great trouble to people’s health and normal social order. The mitigation effect of industrial structure adjustment and technological progress on PM_2.5_ pollution is obvious, but the specific effect has not been quantified. Based on the panel data set of 281 prefecture-level cities in China from 2007 to 2017 and the theory of spatio-temporal dynamics, this paper uses the spatial dynamic panel model to reveal the contribution of industrial restructuring and technological progress to PM_2.5_ pollution. The results show that PM_2.5_ pollution in China has obvious path dependence and spatial diffusion effects. On the whole, industrial restructuring and technological progress have effectively alleviated PM_2.5_ pollution. The decomposition term of technological progress, technical change, has played a significant positive effect on alleviating PM_2.5_ pollution, while efficiency change has played a positive role. Another important finding of this paper is that the combined effect of technical change and industrial restructuring will aggravate PM_2.5_ pollution. 

The empirical research carried out in this paper has multiple significance. First of all, this study confirms that technological progress and industrial structure adjustment have played a positive environmental effect in the prevention and control of PM_2.5_ pollution, which fills the research gap in the field of environmental management to a certain extent. Secondly, the empirical results of this paper can inspire China’s PM_2.5_ pollution control and green development, that is, adhere to the ongoing industrial structure adjustment and innovation incentives at the present stage. Thirdly, China has a vast land area and carries a large population. Alleviating China’s PM_2.5_ pollution is an important part of achieving the goal of global sustainable development. It is of global significance to improve China’s air quality.

### 5.2. Policy Implications

The normal operation of the secondary industry requires a large amount of energy supply, and a large number of fine particles emitted from the production process make the pollution situation of PM_2.5_ worse. A considerable part of the tertiary industry is high-tech industry and service industry, which has far less negative effect on atmospheric environment than the secondary industry. Therefore, the upgrading of industrial structure can achieve positive environmental effects. Rationalization of industrial structure is to realize the adaptation of industrial structure to energy and raw material structure, technical structure and demand structure. The improvement of the rationalization degree of industrial structure provides internal power for reducing emission of particulate pollutants. At the present stage and for a period of time in the future, the Chinese government should not only attach importance to the upgrading of the industrial structure, but also take into account the rationality and coordination of the industrial structure, so as to achieve the sound development of the industry.

Since the implementation of reform and opening up, China has been enjoying the technological dividend brought by trade globalization. In this process, China has carried out a large number of independent innovation activities. At the same time, through the introduction of advanced technology, industrial processes and equipment, China’s technology has been steadily improved in a low-risk and low-cost environment. In other words, China has achieved technological progress in the production process, forming a path of “technological progress-production improvement-pollutant emission reduction”, which has contributed to improving air quality. In recent years, with the narrowing of the technological gap between China and the world powers, more risks are derived from the above path. For example, due to the deepening of capital, if the advanced equipment introduced in the process of industrial development does not match the local resource allocation, the negative environmental effect associated with the above path will dominate, and the combined effect of technological progress and industrial structure adjustment will cause damage to the environment. In the actual process of technology upgrading, attention should be paid to improving the fit between new technologies and resource allocation in order to achieve positive environmental benefits.

Considering the spatial diffusion effect of air pollution, regional joint prevention has profound meaning for alleviating China’s PM_2.5_ pollution. Starting from the top-level design, air pollution control is regarded as an overall and long-term development strategy, and a policy system and governance mechanism from the central to the local level are established. The industrial layout should be rationally planned to avoid excessive concentration of polluting industries and reduce the negative external effects caused by industrial undertaking during industrial transfer. In the process of governance, the cooperation mechanism among different provinces, regions and even cities is formed, and the spatial synergy is formed, and the development concept of green, openness and sharing is established. Promote multi-subjects such as government, enterprises and residents to jointly control PM_2.5_ pollution.

### 5.3. Research Prospects

The relatively rigorous empirical process can simulate the influence mechanism of various factors on PM_2.5_ pollution as much as possible. However, on the one hand, the potential error of available data will make the model results uncertain. On the other hand, the selection of control variables will affect the effect of core variables in the model to some extent. Extensive related research to investigate the mechanism of industrial adjustment and technological progress on the purification of air pollution is an issue that needs to be paid attention to in the future. In addition, the impact of industrial and technical factors on PM_2.5_ pollution has potential regional heterogeneity, which needs to be analyzed deeply in the future.

## Figures and Tables

**Table 1:** Variable statistics results.

Variables	Mean	Std. Dev	Min	Max	Units	Sample Size
PM_2.5_ Concentration (*PM*)	44.63	18.09	3.64	104.30	μg/m^3^	3091
Industrial Structural Upgrading (*ISU*)	37.93	9.43	8.58	80.81	%	3091
Industrial Structure Rationalization (*ISR*)	0.14	1.40	−0.08	50.13	%	3091
Technological Progress (*TP*)	1.00	0.09	0.15	1.94	%	3091
Technical Change (*TC*)	1.01	0.08	0.59	1.31	%	3091
Efficiency Change (*EC*)	1.00	0.09	0.13	1.66	%	3091
Economic Development (*ED*)	41,169.61	28,456.11	3418.00	224,147.00	yuan	3091
Urbanization (*UR*)	49.78	17.89	12.00	98.70	%	3091
Energy Intensity (*EI*)	1.05	0.64	0.08	7.67	t/10^4^ yuan	3091
Temperature (*TE*)	15.15	0.64	1.10	26.80	°C	3091
Precipitation (*PR*)	1006.06	571.28	41.80	3202.50	mm	3091

**Table 2:** Results of global spatial autocorrelation.

Year	I	Z(I)	Year	I	Z(I)
2007	0.583	58.272 ***	2013	0.560	55.926 ***
2008	0.494	49.410 ***	2014	0.529	52.913 ***
2009	0.491	49.125 ***	2015	0.565	56.465 ***
2010	0.531	53.017 ***	2016	0.588	58.720 ***
2011	0.539	53.877 ***	2017	0.499	49.872 ***
2012	0.507	50.692 ***			

Note: The symbols *** indicates *p* < 1%.

**Table 3:** Estimation results based on three models.

	General Dynamic Panel Models		Spatial Static Panel Models		Spatial Dynamic Panel Models	
	Model (*a*)	Model (*b*)	Model (*c*)	Model (*d*)	Model (*e*)	Model (*f*)
*τ*	0.7388 ***	0.7197 ***			0.3271 ***	0.3337 ***
	(0.0407)	(0.0687)			(0.0310)	(0.03184)
*ρ*			0.4639 ***	0.4657 ***	0.4966 ***	0.5008 ***
			(0.0239)	(0.0240)	(0.0195)	(0.0208)
*ISU*	0.0041 *	−0.0542 **	0.0009	−0.0037*	−0.0009	−0.0078 ***
	(0.0029)	(0.0274)	(0.0009)	(0.0027)	(0.0009)	(0.0027)
*ISR*	−0.0226	−2.2674	−0.0015 ***	−0.0794 **	−0.0011 *	−0.1112 ***
	(0.0548)	(2.6874)	(0.0006)	(0.0190)	(0.0006)	(0.0158)
*TP*		−2.0470 **		−0.1801 **		−0.2250 **
		(0.9172)		(0.0887)		(0.0916)
*TP × ISU*		0.0518**		0.0040 *		0.0060 ***
		(0.0260)		(0.0023)		(0.0022)
*TP × ISR*		2.1332		0.0743 ***		0.1047 ***
		(2.5335)		(0.0178)		(0.0151)
*TC*	0.5328 ***		−0.0591 *		−1.1204 **	
	(0.1086)		(0.0320)		(0.0543)	
*EC*	1.2548 ***		0.0216		0.0666 **	
	(0.2211)		(0.0263)		(0.0262)	
ln *ED*	0.0124	0.3708 ***	−0.0758 ***	−0.0779 ***	−0.1064 ***	−0.1082 ***
	(0.0359)	(0.0783)	(0.0143)	(0.0145)	(0.0294)	(0.0293)
*UR*	−0.0039	−0.0076	−0.0022 ***	−0.0021 ***	−0.0010*	−0.0010 *
	(0.0048)	(0.0051)	(0.0007)	(0.0007)	(0.0006)	(0.0005)
ln *EI*	0.0167	0.3049 **	0.0704 ***	0.0732 ***	0.0517 ***	0.0493 ***
	(0.0700)	(0.1296)	(0.0201)	(0.0201)	(0.0148)	(0.0145)
ln *TE*	0.2362 ***	0.2495 ***	0.0958	0.0987	0.1777 *	0.1758 *
	(0.0589)	(0.0789)	(0.0956)	(0.0956)	(0.0933)	(0.0952)
ln *PR*	−0.2280 ***	−1.1394 ***	−0.0248 ***	−0.0244 **	−0.0207 **	−0.0185 *
	(0.0330)	(0.0459)	(0.0092)	(0.0091)	(0.0098)	(0.0097)
Obs	2810	2810	3091	3091	2810	2810
LM–Error			(0.129)	(0.128)	(0.123)	(0.122)
Robust LM–Error			(0.144)	(0.150)	(0.138)	(0.145)
LM–Lag			(0.018)	(0.013)	(0.016)	(0.011)
Robust LM–Lag			(0.037)	(0.025)	(0.032)	(0.020)
AR (1)	(0.000)	(0.000)			(0.000)	(0.000)
AR (2)	(0.798)	(0.356)			(0.472)	(0.307)
Hansen over-Identification test	(0.195)	(0.187)			(0.213)	(0.218)

Note: The symbols *, ** and *** indicate *p* < 10%, *p* < 5% and *p* < 1%, respectively.

## Data Availability

The datasets used and/or analyzed during the current study are available from the corresponding author on reasonable request.
